# Impact of a 10-Week Strength Training Program on Physical Performance and Match External Load in Young Elite Female Soccer Players

**DOI:** 10.3390/jfmk10030289

**Published:** 2025-07-28

**Authors:** Sefika Pelin Bal, Luis Manuel Martínez-Aranda, Peter Krustrup, Javier Raya-González

**Affiliations:** 1 Facultad de Deporte, UCAM Universidad Católica de Murcia, 30107 Murcia, Spain; spbal@alu.ucam.edu; 2 Department of Sports Science and Clinical Biomechanics, SDU Sport and Health Sciences Cluster (SHSC), University of Southern Denmark, 5230 Odense-M, Denmark; pkrustrup@health.sdu.dk; 3 Department of Sports and Computer Sciences, Faculty of Sports Sciences, Universidad Pablo de Olavide, 41013 Seville, Spain; 4 Science-Based Training Research Group (SEJ-680), Physical Performance and Sports Research Center, Universidad Pablo de Olavide, 41013 Seville, Spain; 5 Danish Institute for Advanced Study (DIAS), Faculty of Health Sciences, University of Southern Denmark, 5230 Odense-M, Denmark; 6 Grupo de Investigación en Deporte y Educación Física para el Desarrollo Personal y Social (GIDEPSO), Department of Specific Didactics, Faculty of Education Sciences and Psychology, University of Córdoba, 14004 Córdoba, Spain; rayagonzalezjavier@gmail.com

**Keywords:** resistance training, women, football, team-sport, GPS

## Abstract

Background: Soccer is a physically demanding sport characterized by frequent high-intensity efforts, which are particularly relevant in women’s competitions. Improving high-speed running and aerobic capacity has been linked to better on-field performance. Strength training has shown promise in enhancing these physical attributes, but its application in young female soccer players remains underexplored. This study aimed to investigate the effects of a 10-week in-season strength training program on physical performance and match running demands in young female soccer players. Methods: Thirty-two U18 Danish female professional soccer players from two comparable teams voluntarily participated in the study. Teams were allocated to either an experimental group, performing twice-weekly strength training (EG, *n* = 16) or a control group (CG, *n* = 16). Vertical jump performance and Yo-Yo IR2 performance as an estimation for maximal oxygen uptake (VO_2_max) were assessed both pre and post intervention. Additionally, players’ match external demands (i.e., total distance, distance covered at speeds above 23 km·h^−1^, and maximum velocity achieved) were monitored using Global Positioning System devices during four matches before and after the intervention. Results: Significant within-group differences were observed across all variables for the EG (*p* = 0.001; ES = 1.08 to 1.45, large), without differences in the CG (*p* > 0.01). Between-group analysis indicated significant differences favoring the EG in all variables (F = 27.40 to 47.17; *p* = 0.001). Conclusions: The application of a 10-week strength training program led to improvements in physical and match running performance among young female soccer players, underscoring the importance of incorporating strength training programs into female soccer periodization to enhance performance.

## 1. Introduction

Soccer is a physically demanding intermittent team sport characterized by alternating periods of low- and high-intensity activity [1,2]. Notably, these physical demands differ between men’s and women’s competitions [3], making it essential to analyze them specifically by gender. In women’s soccer, recent research has reported that players cover a total distance of 9 to 10 km per match [4,5], with an average of approximately 30 sprints [6], along with 200 accelerations and 150 decelerations per game [7]. Additionally, female players run around 500 m at speeds exceeding 19.0 km·h^−1^ and 125 m at speeds above 22.5 km·h^−1^ [8]. These findings underscore the importance of enhancing high-intensity capabilities for competitive success [9]. Furthermore, improving maximal oxygen uptake (VO_2_max) is essential for delaying fatigue and supporting repeated high-intensity efforts with minimal recovery time during matches [10]. Therefore, identifying effective strategies to develop these capacities is critical for strength and conditioning coaches.

For this purpose, strength training has been widely recognized as a key factor [11,12]. In this context, the effects of several methodologies have been examined [13], yielding significant improvements in physical performance among soccer players. Specifically, in female soccer, Millar et al. [14] implemented, during the in-season period, a 6-week strength training program focused on back squats in female high school soccer players, achieving significant increases in vertical jump performance. Similarly, Sporiš et al. [15] observed improvements in VO_2_max among female soccer players following a 12-week strength training program during the second off-season period (from November to March). However, soccer presents unique conditions that must be considered when integrating strength training programs into soccer periodization [16]. For example, soccer schedules are often unpredictable and subject to frequent changes, characterizing the sport as highly competitive, with more than one match per week [17]. Additionally, differences between players, such as starters and non-starters, influence the available time for training [18]. These factors must be accounted for to identify the optimal approach regarding frequency, methods, exercises, and session timing. Given the limited time available for implementing specific strength training sessions, it is essential to adopt strategies that enhance performance without inducing excessive fatigue. In this regard, González-Badillo et al. [19] proposed performing half of the maximum possible repetitions for each load during strength training programs. These authors found that this approach promotes faster mean repetition velocities, minimizes neuromuscular performance impairments, facilitates quicker recovery, and reduces hormonal responses and muscle damage compared to completing all possible repetitions. However, this methodology has not yet been applied to young female populations to evaluate its effects on physical performance variables, which is adequate for this population (i.e., young players with scarce experience with resistance training) instead of reaching muscle failure.

Strength and conditioning coaches have emphasized the importance of improving physical performance variables to achieve subsequent increases in distances covered at a high intensity during official matches in the soccer population [20]. In this context, Pedersen et al. [21] demonstrated a strong association between maximal strength, sprint performance, jump height, and match physical performance in high-level female soccer players. Consequently, increases in match load demands contribute to enhanced on-field performance [22]. This is supported by previous research showing that high-standard teams cover greater high-intensity distances during official matches in both male [2] and female soccer players [23]. However, few studies have investigated whether a complementary strength training program can improve soccer players’ performance. In this regard, Byrkjedal et al. [20] confirmed that strength training, applied during the in-season period, is an effective method to enhance external match performance in professional male soccer players, particularly in high-speed running and sprinting variables. Nevertheless, no studies have yet evaluated whether a complementary strength training program for female soccer players could positively impact their match external load variables or used a strength training methodology in which participants complete only half of their maximum possible repetitions, highlighting the need for future research in this area.

To cover this gap, a 10-week strength program was applied in young elite female soccer players during their regular in season. Specifically, the aim of this study was to analyze the effects of a 10-week strength training program on physical performance and match running demands in young female soccer players. Based on prior studies [15,20,24], we hypothesized that a structured training intervention would lead to significant improvements in both physical performance and match running performance among female players in the experimental group compared to a control group.

## 2. Materials and Methods

### 2.1. Study Design

A quasi-experimental design was employed to evaluate the impact of a 10-week strength training program (two sessions per week) on the physical performance and match running demands of young female soccer players. Physical performance was assessed at baseline and post training in a single session, which included a jump test (countermovement jump, CMJ) and an endurance test (Yo-Yo Intermittent Recovery Test). These assessments were conducted on the team’s natural grass training field, with players wearing their own soccer boots. All testing sessions took place in the afternoon between 5:00 p.m. and 7:00 p.m. Additionally, players’ match external demands (i.e., total distance, distance covered at above 23 km·h^−1^, and maximum velocity achieved) were tracked using Global Positioning System (GPS) devices during four matches before and after the intervention period. To standardize conditions, players were instructed to have their last meal 3 h before testing, avoid caffeinated beverages, and refrain from intense physical activity prior to assessments. A strength and conditioning specialist supervised all testing sessions, providing verbal encouragement throughout the protocols [25].

### 2.2. Participants

**Figure 1:**
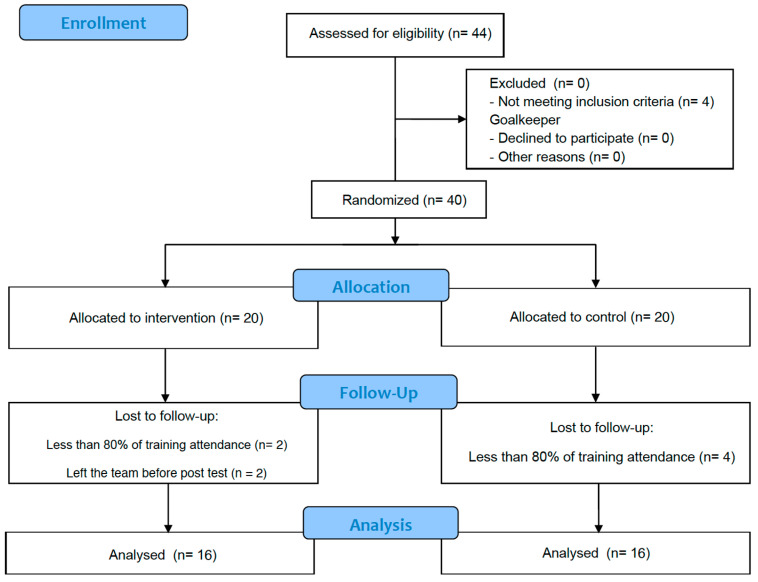
CONSORT diagram of participant’s recruitment, allocation, follow-up, and analysis.

Thirty-two U18 Danish female elite soccer players (age = 16.4 ± 0.8 years) voluntarily participated in the study. A priori power analysis was conducted using G*Power (version 3.1.9.2, Universität Kiel, Kiel, Germany). The analysis indicated that a minimum sample size of 16 participants per group was required to achieve a statistical power (1–3) of 0.80, based on an assumed effect size (ES) of 0.90 (large effect) and a significance level (α) of 0.05. Participants belonged to two different teams competing at the highest level for their age. Both teams followed the same style and training schedule, trained 4 times per week [i.e., first session: recovery or compensation; second session: strength (small-sided games with a reduce number of players); third session: endurance (large-sided games with a high number of players); and fourth session: reaction (medium-sided games with a high number of players)], and played one official match during the weekend. Players were eligible for inclusion in the study if they had been part of the same soccer academy for the past two years, attended at least 80% of training sessions over the 10-week period, had a minimum of four years of experience in systematic soccer training, with low–medium experience in resistance training, and had not sustained any injuries in the two months prior to the study. Teams were randomly assigned to either the control group (CG; *n* = 16; age: 16.4 ± 0.7 years; height: 168.7 ± 5.2 cm; body mass: 62.5 ± 6.4 kg; body mass index: 21.9 ± 1.4 kg·m^−2^) or to the experimental group (EG; *n* = 16; age: 16.4 ± 0.8 years; height: 167.5 ± 5.6 cm body mass: 60.9 ± 6.5 kg; body mass index: 21.7 ± 1.7 kg·m^−2^), with all the players of the same team being parts of each group. Initially, 44 players were recruited for the study, but 4 goalkeepers were excluded due to the distinct nature of their training and role in the game, 2 players left the teams prior to post assessment, and 6 players were injured during the intervention period and did not complete 80% of the training sessions (Figure 1). Prior to participation, all players received detailed information about the study procedures, including potential risks and benefits. Written informed consent was obtained from their parents or legal guardians. The study adhered to the principles of the Declaration of Helsinki (2013) and was approved by the ethical committee of the Catholic University of Murcia under the internal registration number CE062112 (25 June 2021).

### 2.3. Procedures

During the 10-week intervention, players from both groups followed their regular in-season weekly soccer training routine, with the EG incorporating two strength training sessions per week while the CG had no strength training and followed their regular training periodization. The weekly training program (i.e., microcycle) was designed collaboratively by the coach and the strength and conditioning specialist, consisting of four soccer training sessions and one official match. Prior to the intervention, players in the EG completed a 2-week familiarization strength training program. All participants were already accustomed to the testing protocols from routine preseason assessments at the club and were familiar with the GPS devices, having worn them throughout competitive matches during the season. Physical performance assessments were conducted in two testing sessions: Session 1 followed the sequence of the countermovement jump (CMJ) test and the Yo-Yo Intermittent Recovery Level 2 test to minimize fatigue accumulation, while Session 2 included the one-repetition maximum (1RM) assessment. Data from four matches before and four matches after the intervention period were also collected. Before each testing session, a standardized 15 min warm-up was performed. This warm-up included 7 min of slow jogging and walking locomotion, followed by 8 min of jump exercises, progressive acceleration, and sprint drills over 10 and 30 m distances.

### 2.4. Test

#### 2.4.1. Countermovement Jump Height

Players completed two bilateral countermovement jumps (CMJs) with 1 min of rest between attempts. They were instructed to perform a downward motion followed by a rapid and complete extension of the lower limbs to maximize jump height, keeping their hands on their hips throughout the movement. The MyJump 2.0 mobile application was used to measure CMJ height, a tool shown to be highly valid (r = 0.995) and reliable (intraclass correlation coefficient = 0.997) for this purpose [26]. All jumps were recorded at 240 Hz with an iPhone 8 Plus mobile device (Apple Inc., Cupertino, CA, USA) [27]. The best jump height (in cm) was selected for further analysis.

#### 2.4.2. Yo-Yo Intermittent Recovery Level 2 Test

A single set of this test was completed, which involved performing 2 × 20 m shuttle runs at progressively increasing speeds, with 10 s of active recovery between runs. The recovery consisted of jogging 2 × 5 m. Players followed an auditory beep signal, and the test concluded when they failed to reach the finish line in sync with the beep on two consecutive attempts. The total distance covered (in m) was recorded as the test result [28]. From this test, each player’s estimated VO_2_max was calculated indirectly. The test area was marked with cones, forming a 20 m long and 2 m wide running lane. An additional cone, placed 5 m behind the finish line, indicated the jogging distance for the active recovery period.

### 2.5. Match Running Demands

The Polar Team Pro (Polar Electro, Kempele, Finland) device was used to monitor match external demands. It is an advanced athlete monitoring system designed for team sports, integrating a 10 Hz GPS, 200 Hz tri-axial accelerometer, gyroscope, magnetometer, and heart rate monitor. These devices were positioned on the chest of players, with each player using the same device during the entire experimental period. These devices have shown high accuracy and acceptable reliability compared to the gold standard [29]. Specifically, the following variables were monitored: total distance covered in meters (TD), very-high-speed running distance (VHSR, >23 km·h^−1^), and maximum velocity achieved during matches (Vmax).

### 2.6. Strength Training Program

Players participated in a 10-week complementary strength training program (CSTT), conducted prior to regular soccer training and following a standardized warm-up. The CSTT incorporated strength, range of motion (ROM), and balance exercises, as detailed in Table 1 and Table 2. Strength exercises targeting major muscle groups (e.g., chest, legs, back) were performed at half of the maximum number of repetitions per set, with 6 repetitions of the possible 12 [70% 1RM load (4 × 6 (12))]. This approach was based on evidence from a prior study, conducted with physically active sports science students, demonstrating that performing half the maximum number of repetitions per set promotes faster mean repetition velocities, minimizes neuromuscular performance impairments, facilitates faster recovery, and reduces hormonal responses and muscle damage following exercise [19]. An incremental load test was conducted to determine the 1RM value for each exercise [30]. The initial load for all participants was set at 20 kg and was progressively increased by 10 kg per set until reaching 0.5 m/s in mean propulsive velocity. Subsequently, the load was increased by 5 to 2.5 kg until the participant could no longer move it. A rest period of at least 5 min was allowed between sets. A re-evaluation was implemented prior to the sessions of the 6th week. Balance exercises were performed on Bosu, with players having to reach 3 movement directions with their hand, completing 3 sets per exercise. Players completed Session 1 (Table 1) during their first strength training session of the week and Session 2 (Table 2) during their second strength training session. Prior to the pre-test session, players underwent a 2-week anatomical adaptation phase (2 sessions per week). Throughout the 10-week in-season intervention period, both CG and EG players adhered to their regular in-season routines, comprising 4 weekly 60–90 min training sessions in addition to competitive matches.

### 2.7. Statistical Analysis

Descriptive statistics are presented as means ± standard deviations (SDs). The Shapiro–Wilk test was conducted to assess the normality of the data distribution, and Levene’s test was utilized to confirm the homogeneity of variances. To detect between-group and within-groups differences, an analysis of covariance (ANCOVA), incorporating baseline values as covariates, was performed. When significant *F* values were found, Bonferroni post hoc analysis was performed. Effect sizes (ESs) were computed using Cohen’s method to evaluate the magnitude of effects and were interpreted as follows: <0.2, trivial; 0.20–0.49, small; 0.50–0.80, moderate; and >0.80, large [31]. Statistical analyses were carried out using SPSS v29 (SPSS Inc., Chicago, IL, USA). Based on the Bonferroni correction, statistical significance was set at *p* < 0.01.

## 3. Results

No significant between-groups differences were found when baseline values were compared.

**Figure 2:**
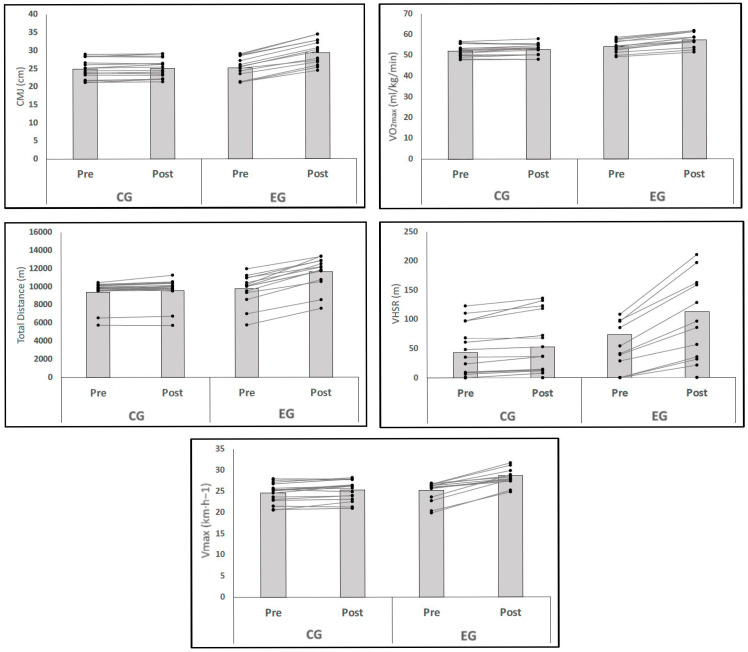
Individual changes in physical performance and match load variables before (baseline) and after (post training) the 10-week intervention period. Abbreviations: CG = control group; EG = experimental group.

The changes in physical performance and match load variables before (baseline) and after (post training) the 10-week intervention period are presented in Table 3 and Figure 2.

In the within-group analysis, the experimental group (EG) exhibited substantial improvements in all variables (*p* = 0.001), with large effect sizes (ESs). CMJ performance increased by 16.33% (ES = 1.44), VO_2_max improved by 5.80% (ES = 1.13), and TD showed a marked increase of 19.39% (ES = 1.18). Moreover, VHSR almost doubled, with an increase of 96.55% (ES = 1.08), and Vmax improved by 13.47% (ES = 1.45), highlighting the considerable magnitude of the training effects in the EG. Conversely, no within-group differences were observed in the CG for any variable.

Between-group comparisons revealed significant differences in favor of the EG across all variables (*p* = 0.001). The CMJ showed the largest group difference, followed closely by VO_2_max and TD. VHSR and Vmax also demonstrated substantial between-group effects, confirming the superior improvements in physical performance and match load performance in the EG compared to the CG.

## 4. Discussion

This study aimed to examine the effects of a 10-week strength training program on physical performance and match running performance in young female soccer players during their regular in-season period. The CG did not perform any strength training, as it was not included in their in-season periodization program. To the best of our knowledge, this is the first study to investigate the impact of strength training based on completing half of the maximum number of repetitions per set on match load performance in this population (i.e., female elite U-18 soccer players). The main obtained findings indicated that players in the EG achieved significantly greater improvements compared to those in the CG.

The CMJ is widely recognized by soccer strength and conditioning professionals as a reliable measure of lower extremity power [32], which is strongly associated with success in soccer [9]. Consequently, training programs should prioritize improving this parameter. In this study, increases in CMJ performance were observed in the EG following the intervention period, with significant between-group differences in favor of this group. These findings align with those of Millar et al. [14], who reported a 4.9% improvement in vertical jump performance after implementing a 6-week in-season strength training program focused on back squats in female high school soccer players. Similarly, Lambright et al. [24] conducted an 8-week strength training program (three sessions/week) during the preseason in young female soccer players (i.e., 16 years), observing improvements of 1.19 ± 2.71 cm in CMJ performance post intervention. The training group also seemed to improve the estimated VO_2_max, potentially derived from an improvement in the muscles’ ability to use oxygen. Enhancements in VO_2_max contribute to delaying the onset of fatigue, thereby enabling repeated high-intensity efforts with minimal recovery time during matches [10]. In this study, an estimated improvement in VO2max were observed in the EG (5.8%), although it cannot be ruled out that the improvement in Yo-Yo IR2 performance, was related to improvements in muscle strength and power. Similar findings have been reported in previous research. For example, Sporiš et al. [15] documented increases in VO_2_max following a 12-week strength training program (three sessions/week) during the off-season period in elite female soccer players (under 20 years). These results highlight the importance of incorporating strength training programs into soccer periodization to enhance both high-intensity actions and aerobic power. Also, it is necessary to highlight that these strength training programs could be enhanced if their methodology is based on performing half of the reps in the main lifts, since although prior studies presented significant improvements, in the present study, these improvements were also achieved but in a more time-efficient manner (i.e., half the reps), while generating less labor that could interfere with soccer performance.

Certain external load metrics, such as TD, VHSR, and Vmax achieved during matches, are critical determinants of soccer performance, with top-tier teams often exhibiting the highest values in these variables [2]. Consequently, it is essential to develop strategies that enhance performance in these metrics during matches to achieve greater soccer success. In this study, the between-group comparison revealed significantly greater improvements in the EG for TD, VHSR, and Vmax. These findings may be attributed to the strong relationship between strength levels and high-intensity actions, as well as external match performance [21,33]. Also, we hypothesize that the applied strength training could improve sprint mechanics, support higher maximum velocities, potentiate repeated-sprint ability, enable more very-high-speed running bouts, and delay neuromuscular fatigue, facilitating greater total distance covered during match play. Despite this, no previous studies have specifically examined the impact of strength training programs on external match load performance in young female soccer players, although this has been explored in men’s soccer. For example, Byrkjedal et al. [20] implemented two distinct strength training programs (i.e., regulated vs. self-selected) over a 10-week period (one session per week) in professional male soccer players. Both groups demonstrated improvements in external match load variables, such as high-speed running and sprint running. These findings reinforce the notion that strength training is a crucial component for enhancing external match performance during official competitions.

Despite the promising findings, this study has several limitations that practitioners should consider. The main limitation is that two teams were involved, and all the players of the same team were allocated into the same group. In this sense, although the training standards of both groups were similar, this could have impacted the results obtained due to the influence of the coaches. Another limitation is that the EG completed a higher total training volume, as the CG did not supplement their regular soccer training. Also, only two teams participated in the study, consistently maintaining the same roles. Therefore, future research involving a greater number of teams or employing a crossover design is needed to draw more robust conclusions. In addition, the assumed GPS variability must be considered, mainly when high-intensity distances are assessed. Finally, other important abilities, including flexibility, change in direction speed, and repeated-sprint ability (RSA) must be assessed in future studies. Finally, the study exclusively included female soccer players, which limits the generalizability of the findings to male players, highlighting the need for similar studies in male populations.

## 5. Conclusions

The main findings in this study showed improvements in young female soccer players’ physical performance and match running performance after the application of a 10-week strength training program. Using a practical approach, it is suggested to incorporate the proposed strength training program, focused on the lower limb, into young female soccer players’ periodization to improve not only physical performance but also match load parameters. Future studies considering the impact of the strength program on injury occurrence or long-term studies analyzing the retention of performance gains should be performed.

## Figures and Tables

**Table 1:** Session 1 for the complementary strength training program.

Exercises	Illustrations		Sets	Repetitions
Bulgarian split squat	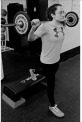	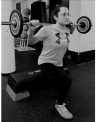	4	6 (12)
Quick steps with band	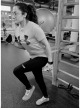	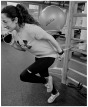	4	10 s
Dumble bent row	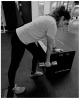	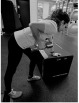	4	6 (12)
One leg deadlift	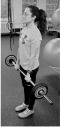	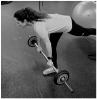	4	6 (12)
Quick step up	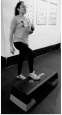	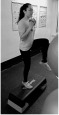	4	6 each leg
Lever chest press	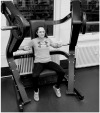	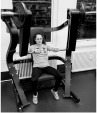	4	6 (12)
Cable abduction + adduction	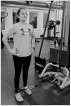	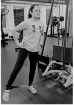	2	6 (12)
	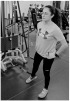	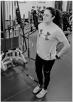		
Reverse hyperextension	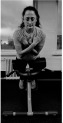	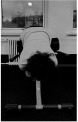	3	12
Knee up crunch + leg raise	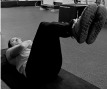	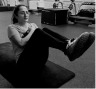	3	15 + 10
	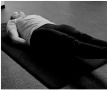	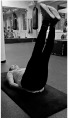		
Plank series	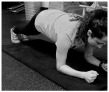	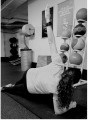	3	30 s + 10 s + 30 s + 10 s
	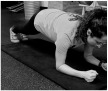	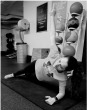		
One leg on Bosu, reaching 3 directions with each hand	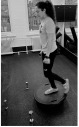	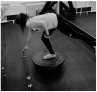	3	One set each leg
	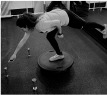	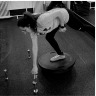		

Notes: 6 (12) = to perform 6 reps of 12 possible reps; body weight exercises did not follow the half rep method.

**Table 2:** Session 2 for the complementary strength training program.

Exercises	Illustrations		Sets	Repetitions
Walking lunge	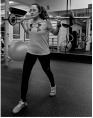	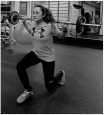	4	6 (12)
	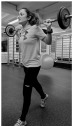	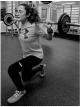		
Bird dog	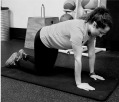	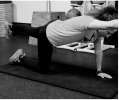	3	12 each side
4-sided change of direction CMJ and drop jump	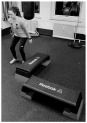	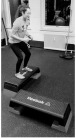	3	3 rounds
	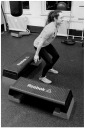	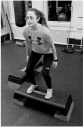		
Prone hamstring curl	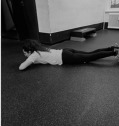	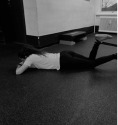	4	6
Leg raise and hip raise	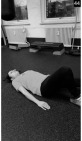	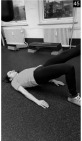	3	6 + 6
Boxing	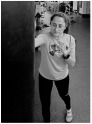	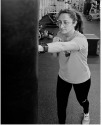	3	1 min
Abduction + adduction with band	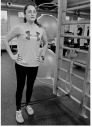	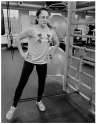	2	12
	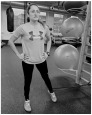	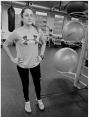		
V-sit trunk rotation with weight	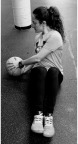	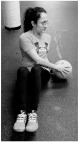	3	6 each side
One-leg side-to-side jump and hold	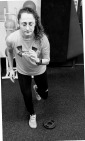	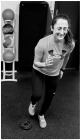	4	5
Plank series	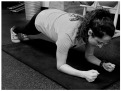	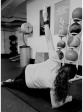	3	30 s + 10 s + 30 s + 10 s
	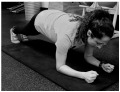	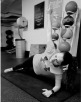		

Notes: 6 (12) = to perform 6 reps of 12 possible reps; body weight exercises did not follow the half rep method.

**Table 3:** Changes in physical performance and match load variables before (baseline) and after (post training) the 10-week intervention period.

	CG (n = 16)					EG (n = 16)					Between Group Differences		
Variables	Baseline (Mean ± SD)	Post Training (Mean ± SD)	Δ (%)	p	ES	Baseline (Mean ± SD)	Post Training (Mean ± SD)	Δ (%)	p	ES	F	p	η^2^
CMJ (cm)	24.84 ± 2.72	25.07 ± 2.65	0.93	1.000	0.08	25.10 ± 2.84	29.20 ± 3.37	16.33	0.001	1.44	45.75	0.001	0.302
VO_2_max (ml/kg/min)	52.03 ± 2.87	52.51 ± 2.83	0.92	0.342	0.17	54.01 ± 2.78	57.14 ± 3.22	5.80	0.001	1.13	47.17	0.001	0.150
TD (m)	9377 ± 1300	9543 ± 1370	1.77	0.675	0.13	9773 ± 1566	11628 ± 160.70	19.39	0.001	1.18	31.59	0.001	0.250
VHSR (m)	42.88 ± 44.17	52.67 ± 49.93	22.83	0.694	0.22	47.00 ± 41.90	92.38 ± 71.90	96.55	0.001	1.08	27.40	0.001	0.074
Vmax (km·h^−1^)	24.63 ± 2.35	25.24 ± 2.39	2.48	0.498	0.26	25.17 ± 2.33	28.56 ± 2.20	13.47	0.001	1.45	38.26	0.001	0.294

Abbreviations: CG = control group; EG = experimental group; SD = standard deviation; Δ (%) = percentage of change between pre- and post-intervention values; *p* = level of significance; ES = effect size; CMJ = countermovement jump; VO_2_max = maximal oxygen uptake; TD = total distance; VHSR = distance covered at above of 23 km·h^−1^; Vmax = maximum velocity achieved during matches. Significance level was set at *p* < 0.05.

## Data Availability

The data are available upon request to the corresponding author.
